# Preclinical Research on Focused Ultrasound-Mediated Blood–Brain Barrier Opening for Neurological Disorders: A Review

**DOI:** 10.3390/neurolint15010018

**Published:** 2023-02-14

**Authors:** Chanho Kong, Won Seok Chang

**Affiliations:** Department of Neurosurgery, Yonsei University College of Medicine, Seoul 03722, Republic of Korea

**Keywords:** focused ultrasound, blood–brain barrier, neurological disorders, drug delivery

## Abstract

Several therapeutic agents for neurological disorders are usually not delivered to the brain owing to the presence of the blood–brain barrier (BBB), a special structure present in the central nervous system (CNS). Focused ultrasound (FUS) combined with microbubbles can reversibly and temporarily open the BBB, enabling the application of various therapeutic agents in patients with neurological disorders. In the past 20 years, many preclinical studies on drug delivery through FUS-mediated BBB opening have been conducted, and the use of this method in clinical applications has recently gained popularity. As the clinical application of FUS-mediated BBB opening expands, it is crucial to understand the molecular and cellular effects of FUS-induced microenvironmental changes in the brain so that the efficacy of treatment can be ensured, and new treatment strategies established. This review describes the latest research trends in FUS-mediated BBB opening, including the biological effects and applications in representative neurological disorders, and suggests future directions.

## 1. Introduction

**Figure 1:**
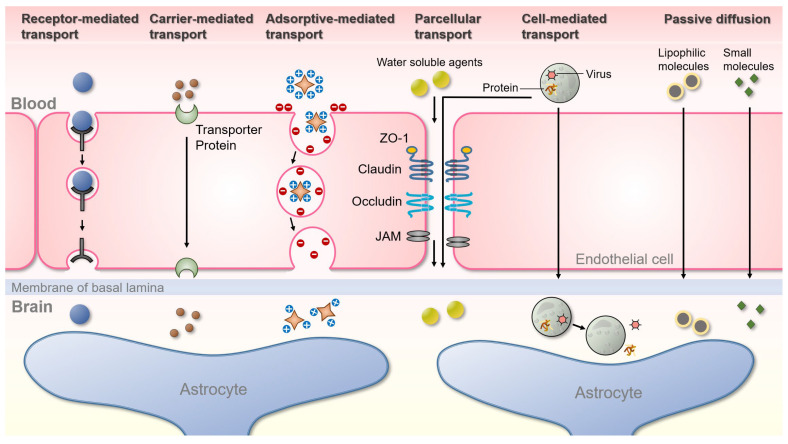
Schematic representation of the various pathways for transport across the BBB. Receptor-mediated transport (Clathrin-mediated endocytosis): only receptor-specific substances are transported through this process, and cells take up metabolites, hormones, proteins, and in some cases, viruses by an internal invasion of the plasma membrane. Carrier-mediated transport is an energy-dependent pathway normally used by small hydrophilic molecules. Carrier membranes have specific receptors that recognize target molecules and pass through cells, and mainly amino acids, monosaccharides, and peptides are delivered in this process. Adsorptive-mediated transport: this is accomplished by the electrostatic interaction of negatively charged plasma membrane with oppositely charged ligands. Paracellular-mediated transport is a passive transport process across the epithelium through the intercellular space between endothelial cells, in which various tight junction proteins are intricately attached. Cell-mediated transcytosis: cells such as monocytes or macrophages migrate through the paracellular space or across the BBB by transcytosis into the brain to release specific proteins or viruses. Passive diffusion: most small molecules cross the BBB and reach the brain by passive diffusion.

Neurological disorders are medically defined as diseases that affect the brain and the nerves throughout the central and peripheral nervous systems. There are more than 600 neurological disorders, including degenerative diseases, such as Alzheimer’s or Parkinson’s; brain tumors; brain or spinal cord injury; convulsive disorders, such as epilepsy; and diseases of the blood vessels that supply the brain, such as stroke. However, the etiology of many central nervous system diseases has not yet been identified, and various new drugs are being developed accordingly. However, unlike other organs, the brain that oversees the central nervous system (CNS) is protected by a blood–brain barrier (BBB). The BBB separates the lumen of cerebral blood vessels from the brain parenchyma and selectively restricts permeation through tight junctions between vascular endothelial cells. Each of these tight junctions is composed of a protein complex of various transmembrane proteins, such as junctional adhesion molecules (JAM), occludin and claudin (Figure 1). Outside of the BBB, it forms a structure with astrocytes and pericytes. In particular, the astrocytic endfeet establish connections between neurons and blood flux and regulate the formation of the BBB [1]. The BBB regulates the homeostasis of the CNS by forming a special structure that prevents exogenous compounds and harmful or toxic substances from being delivered into the brain via the cerebral blood vessels. However, the BBB also limits the intra-brain delivery of various medications. Currently, many brain disease treatments are being developed, but 100% of the drugs with large molecules and 98% or more with small molecules cannot cross the BBB [2].

Meanwhile, dysfunction of the BBB is a critical factor in various diseases such as epilepsy and stroke [3]. Reportedly, the BBB is broken down in neurodegenerative brain diseases such as Alzheimer’s disease (AD), but the role of this phenomenon is unclear [4]. In addition, malignancies damage the BBB through the formation of the blood–tumor barriers (BTBs) [5], and although BTBs leak more than the BBB [6], there remain limitations in drug delivery due to heterogeneous permeabilities and efflux transporters [7,8]. In order to pass through the luminal membrane of brain endothelial cells that consist of the BBB, a number of substances in the blood act on various metabolic enzymes or are actively released into the capillary lumen by embedded efflux transporters such as permeability-glycoprotein (Pgp). Pgp is a protein present in the plasma membrane of endothelial cells in the BBB and one of several efflux pumps. Pgp is overexpressed not only in the selectively permeable BTB, but also in the plasma membrane of tumor cells, which makes tumors cross-resistant to other anticancer drugs [9,10]. Therefore, a technique that selectively inhibits efflux transporters such as Pgp in the target region is needed. Tumor treatment strategies using FUS are highly important, given that FUS-mediated BBB opening not only affects vesicular transcellular transport, but also inhibits Pgp expression.

Several pathways can cross the BBB to maintain brain homeostasis. Representative pathways include paracellular, transcellular, carrier-mediated, receptor-mediated, adsorptive-mediated, and cell-mediated pathways [11] (Figure 1). To date, numerous therapeutic strategies have been developed to overcome the BBB. The main method involves the transcellular lipophilic pathway, but many drugs are hydrophilic; therefore, the efficacy of this pathway is restricted. The method of changing the tight junction using mannitol, an osmotic diuretic, is an example of a paracellular pathway, and it is not useful enough to be applied clinically [12]. Recently, strategies to overcome the BBB through the development of carriers such as liposomes, nanoparticles, viruses, and exosomes, have been attempted. However, they face limitations in terms of safety and efficiency [13].

**Figure 2:**
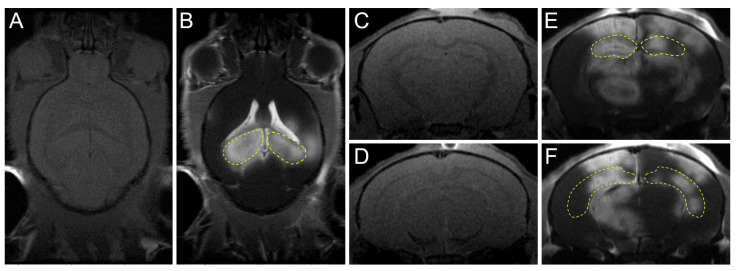
Confirmation of FUS-induced BBB opening using MRI. The hippocampi (yellow dotted line) of mouse were targeted per sonication. (**A**,**B**) Transverse T1-weighted pre-/post-gadolinium MR images were taken to confirm the increased BBB permeability. (**C**–**F**) Coronal T1-weighted pre-/post-gadolinium MR images after FUS. (**E**,**F**) Coronal T1-weighted post-gadolinium MR images after FUS.

**Figure 3:**
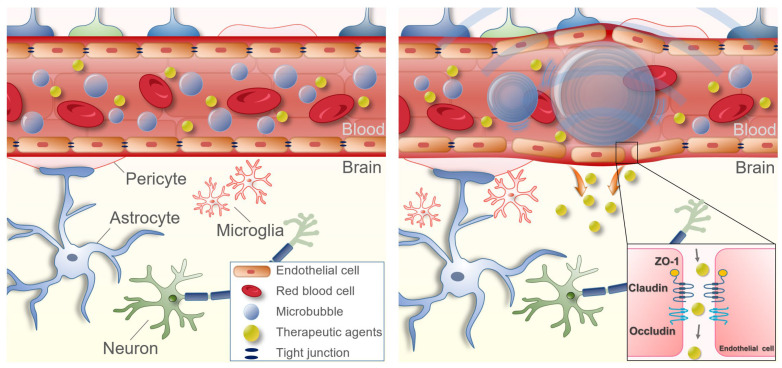
Schematic representation of focused ultrasound-mediated focused blood–brain barrier opening. When FUS is sonicated in a specific area of the brain, an acoustic cavitation effect is induced, in which MB injected into the blood repeats contraction and expansion due to the pressure of FUS. The binding force between the vascular endothelial cells is loosened at this time. The loosened binding force lasts about 6 h, during which drugs can be delivered into the brain parenchyma.

An invasive surgical method, in which the skin is incised and the skull is opened to, has been used for a long time to treat brain diseases. However, minimally invasive or non-invasive surgical methods are being developed to avoid various risks associated with operating on a larger target area, such as functional damage to the brain and infection. Focused ultrasound (FUS) enables a superior penetration depth and spatial specificity without invasive surgical procedures or genetic modifications. Low-intensity FUS with microbubbles (MB) is a non-invasive technique that reversibly and temporarily opens the BBB [14,15]. Since contrast agents cannot pass through the BBB, FUS-induced BBB opening is usually confirmed using contrast-enhanced MRI (Figure 2) [16]. Although the mechanism underlying the effect of BBB opening using FUS has not been elucidated, it is generally thought that the physical oscillations caused by the MB affect the vascular endothelial cells and tissues (Figure 3). In one study, intravenous injection of MB followed by sonication of a specific area of the brain by FUS led to an acoustic cavitation phenomenon wherein the MB repeatedly contracted and expanded in the treated area [17]. The physical BBB opening returns to normal approximately 6 to 24 h after sonication [18]. In the past 20 years, drug delivery studies have been conducted for various diseases through FUS-mediated BBB opening. This review describes the latest research trends in FUS-mediated BBB opening.

## 2. Current Status of FUS-Mediated BBB Opening

### 2.1. Alzheimer’s Disease

The incidence of Alzheimer’s disease (AD), the most representative neurodegenerative brain disease, is steadily increasing as the aging population increases. However, only drugs that can alleviate and delay symptoms are currently being used, and no specific treatment methods or therapeutic agents [19] have been developed yet. Over the past decades, several clinical trials have been conducted with various targets, focusing on these two clinical indications: amyloid beta plaques and neurofibrillary tau tangles [20]. However, all clinical trials have failed; only Aducanumab, which targets amyloid-β (Aβ) plaque removal, has shown a therapeutic effect, but it is controversial due to side effects [21,22]. Although the amyloid hypothesis remains controversial, since the accumulation of Aβ is a representative pathological hallmark of AD, numerous therapeutic studies targeting Aβ have been conducted.

The first preclinical study on FUS for AD aimed to deliver anti-Aβ antibodies targeting amyloid plaques into the brain by a BBB opening. Consequently, anti-Aβ antibodies bound to the Aβ plaques and rapidly reduced the plaque pathology [23]. Subsequently, research on delivering therapeutic agents through FUS-mediated BBB opening in patients with AD has gained attention [24,25,26,27,28,29,30]. Interestingly, studies have reported that amyloid pathology [24,31,32,33,34] and phosphorylated tau [35,36] are reduced only by FUS-induced BBB opening without specific drug delivery. Treatment delivery via FUS-mediated BBB opening also affected memory recovery in AD animal models [32,37,38,39,40]. Research studies on various biological changes by FUS-mediated BBB opening are ongoing. However, for FUS to be a promising non-pharmacological treatment delivery method for AD, further research is needed on why amyloid is reduced and cognitive function is restored. FUS induces the activation of microglia and astrocytes, which may increase phagocytosis of the amyloid plaques [31,32,41]. Recently, a study confirming the therapeutic effect in an AD mouse model (5×FAD) by combining FUS and Aducanumab was reported [42]. Aducanumab, a monoclonal antibody targeting fibril forms and beta-amyloid oligomer, has been proven effective since receiving FDA approval in 2021. However, due to side effects, debate continues as to whether or not it should be used.

In conclusion, combined treatment with FUS and Aducanumab reduced amyloid plaque levels, increased hippocampal neurogenesis, and restored cognitive function. Here, FUS activated phagocytic microglia and increased the number of astrocytes associated with amyloid plaques. This suggests that FUS can induce a reduction in amyloid plaques through phagocytosis. In addition, an RNA sequencing analysis showed that the combined treatment with FUS and Aducanumab upregulated neuroinflammation signaling, phagosome formation, reelin signaling, and CREB signaling [42]. The immunomodulatory effect of FUS, such as the activation of various innate immune cells, plays a vital role in reducing amyloid plaques [37]. Regarding the recovery of cognitive function by FUS, the increase in hippocampal neurogenesis [43,44,45,46] or synaptic plasticity [45,47] may play a role here, but further research is needed on this topic. We summarized the most relevant preclinical studies on FUS-mediated BBB opening in AD (Table 1).

### 2.2. Parkinson’s Disease

Parkinson’s disease (PD) is a neurodegenerative brain disease accompanied by motor dysfunction due to the loss of dopaminergic neurons. PD is neuropathologically characterized by proteinaceous inclusions called Lewy bodies [56]. Notably, as many studies have reported that α-synuclein plays a direct role in disease development, PD is classified as α-synucleinopathies [57]. Currently, there are no clear treatments to slow or alleviate the progression of neurodegenerative diseases such as PD. Treatment with glial-derived neurotrophic factor (GDNF) is considered appropriate for PD due to its neuroprotective and neurotrophic effects [58,59,60]. The overexpression of neuroprotective genes that induce dopamine regeneration in activated neurons can delay disease progression [61]. The potential benefit of GDNF with regard to recovery and the functional improvement of dopaminergic neurons has been confirmed [61,62,63]; however, one study was discontinued due to safety concerns in clinical trials [64]. Animal studies of GDNF gene delivery by FUS began in PD and have highlighted the possibility of effective gene therapy [65,66,67].

Since neurturin has been found to have neuroprotective and neuro-regenerative effects on dopaminergic neurons [68], the FUS-based delivery of neurturin has been studied to find an alternative to GDNF [69,70]. Recently, recombinant adeno-associated viral (rAAV) vectors have received much attention as a tool for gene delivery to the brain. The technology of delivering rAAV using FUS-mediated BBB permeability and expressing the delivered gene has already been examined [71]. Accordingly, recent studies on PD models using FUS mainly involve gene delivery using rAAV. While there are many studies on delaying disease symptoms by delivering various therapeutic agents using FUS, there is a lack of preclinical research studies on α-synuclein-based PD models. We summarized the most relevant preclinical studies on FUS-mediated BBB opening in PD (Table 2).

### 2.3. Brain Tumor

Glioblastoma is the most aggressive brain tumor with a high recurrence rate and poor prognosis despite treatments such as resection, radiotherapy, and chemotherapy [77]. The blood–tumor barrier (BTB) is created by the often heterogeneous disruption of the BBB within the tumor due to aberrant angiogenic signaling. As the delivery of anticancer drugs is limited despite the irregular leakiness of the BTB, quantitative drug delivery through FUS-mediated BBB opening is required [78]. Many previous studies on drug delivery by FUS have involved patients with brain tumors. Doxorubicin is a chemotherapeutic agent that inhibits cell growth and induces apoptosis in malignant glioma cells; however, it is not commonly used because it cannot cross the BBB. In 2007, Treat et al. delivered doxorubicin to a tumor in the brain via FUS-mediated BBB opening, indicating that this drug could be a viable treatment option [79]. Until now, various therapeutic agents have been used to treat glioblastomas, and FUS-mediated BBB opening technology is being developed. In the early days of FUS research, unencapsulated drugs such as the common anticancer drug temozolomide (TMZ) [80,81], carmustine (BCNU) [82], and immunostimulatory interleukin-12 (IL-12) [83,84] were mainly used.

Brain metastasis represents an important predictor of mortality for various non-brain cancers such as breast cancer. Like primary brain tumors, brain metastases do not have an intact BBB, but most therapeutics still have lower intra-tumoral bioavailability than non-brain tumors [85]. FUS studies have continued to treat metastatic brain tumors as well as primary brain tumors. In 2012, there was a study confirming the therapeutic effect by delivering Trastuzumab based on FUS-BBB opening in a breast cancer brain metastases model [86]. Additional research reported in 2016 demonstrated that the administration of trastuzumab and pertuzumab in a brain metastasis mouse model of breast cancer inhibited the growth of brain metastasis when used with FUS, compared to chemotherapy alone [87].

Whether it is a primary brain tumor or a metastatic brain tumor, the critical factor in the tumor microenvironment is to what extent the anticancer drugs could be delivered into the target region. It has been reported that the delivery of chemotherapeutic agents with small molecular weights to the brain tumor microenvironment is approximately 3.9-fold higher under FUS-mediated BBB opening conditions [88]. This enhanced delivery rate has been shown to increase median survival by approximately 30% compared to chemotherapy alone.

However, efflux transporters such as Pgp are overexpressed in cancer cells and prevent the uptake of anticancer drugs into the cells, resulting in resistance to them. FUS-mediated BBB opening temporarily inhibits Pgp expression, thereby preventing drug efflux and interfering with functional components of the BBB [89]. Additional research is needed on efflux transporter inhibitors targeting cancer cells. In addition to unencapsulated drugs, studies have reported that tumors (metastatic breast cancer) can be effectively controlled by delivering natural killer cells under BBB opening [90]. Furthermore, studies on suppressing brain tumors by delivering patient-specific antibodies or complexes loaded on short-hairpin RNA-liposomes have also been previously reported [91]. Since then, several studies have been conducted to enhance the safety and efficiency of tumor treatment by delivering encapsulated therapeutics through the conjugation of existing drugs or genes with improved MB, virus, and nanoparticles [92,93,94,95]. As immunotherapy is a critical issue in neuro-oncology, additional research on immunotherapy using FUS-mediated BBB opening is expected to become more active in the future. We summarized the most relevant preclinical studies on FUS-mediated BBB opening in brain tumors (Table 3).

## 3. Secondary Biological Effects

### 3.1. Neurogenesis

In 2014, Scarcelli et al. first reported that FUS-meditated BBB opening significantly increased the number of proliferating cells and newborn neurons in the dentate gyrus of the hippocampus [43]. Since then, FUS has been considered a therapeutic strategy to improve learning and memory in patients with neurological disorders such as AD, thus going beyond a tool for drug delivery. Neurogenesis is induced under conditions involving BBB opening within appropriate parameters, but not FUS stimulation without MB [46]. The fact that FUS-mediated BBB opening induces neurogenesis has been proven in many studies [28,44,103]. In our previous study, adult hippocampal neurogenesis was induced after 18 days of FUS treatment, and BDNF and early growth response protein-1 were upregulated [44].

In addition, studies have recently reported that the regulation of ERK signaling cascades is involved in neurogenesis after BBB opening. However, it is necessary to understand the specific mechanism underlying FUS-induced neurogenesis. FUS can be a non-pharmacological therapeutic strategy for treating neurodegenerative brain diseases in older patients or patients with AD who have decreased hippocampal neurogenesis [104,105].

### 3.2. Glymphatic System

The glymphatic system is a unique fluid transport system of perivascular channels formed by astroglial cells to facilitate the efficient clearance of soluble proteins and metabolites from the CNS [106]. Damage to the glymphatic system is closely related to several neurological diseases, such as AD, PD, stroke, and traumatic brain injury [107,108,109,110,111]. Conversely, since improvements in an impaired glymphatic system can alleviate these diseases, attempts have been made to find ways to improve the glymphatic system [112,113,114,115].

FUS-mediated BBB opening without any drug delivery in the AD model reduced the amyloid pathology, improved cognitive function, and increased the phagocytosis of glial cells [32,34,50]. Since the glymphatic system can promote the removal of pathological proteins such as amyloid plaques, it is necessary to study the amyloid plaque reduction effect of FUS-mediated BBB opening. According to related research results, FUS-meditated BBB opening increases brain-to-CSF Aβ drainage and induces glymphatic–lymphatic reduction in Aβ [116]. Previously, Meng et al. investigated the accumulation of MRI contrast agents in the draining vein and subarachnoid space after FUS-meditated BBB opening in the human brain [117]. Ye et al. also reported that FUS-mediated BBB opening could enhance glymphatic transportation in the brain [118].

It is not confirmed whether the glymphatic system studied in rodents also exists in humans [119]. This is because there are no human studies that have characterized the flow of this system. In sum, since the activation of the glymphatic system through FUS can induce the clearance of various harmful proteins, such as Aβ or α-synuclein, specific additional studies on the glymphatic system are needed. In addition, the cerebral blood flow and lymphatic systems are structurally and functionally different between humans and rodents. Therefore, research involving the visualization of the blood flow or glymphatic–lymphatic system needs to be conducted in mammals, starting with primates.

### 3.3. Inflammatory Response

One of the secondary effects beginning within hours after FUS-mediated BBB opening is inflammatory responses. Inflammatory responses are biological responses to harmful stimuli and act as a defense mechanism involving immune cells, blood vessels, and inflammatory mediators. Microglia are one of the basic innate immune cells in the brain. Studies have reported that microglia activation occurs 1, 6, and 24 h after FUS-mediated BBB opening [120,121]. Further, as mentioned in Section 2.1 Alzheimer’s Disease, a study reported that FUS and aducanumab combined treatment in an AD mouse model reduced amyloid plaques. At this time, increased microglia and astrocytes were suggested to reduce plaques through the phagocytosis effect [42]. Similarly, studies were also reported confirming that the immunoreactivity of resident Iba1+ and phagocytic CD68+ microglial cells and a transient increase in the infiltration of Ly6G+ immune cells increased 4 and 72 h after FUS-BBB opening [54].

Recently, research on various changes after FUS-mediated BBB opening has been explored using sequencing techniques such as transcriptomics and proteomics [122,123,124]. McMahon et al. showed that many pro-inflammatory genes were upregulated, and BBB transporter genes were down-regulated 6 h after BBB opening, which returned to baseline within 24 h. However, angiogenesis-related genes were upregulated at 6 and 24 h [125]. McMahon et al. also emphasized the importance of the optimization of FUS parameters because FUS induces BBB opening regardless of the upregulation of the NFκB signaling pathway, although a damaging inflammatory response was detected at high MB doses [122]. Recently, Ji et al. investigated changes in the relative gene expression of mouse inflammatory cytokines and receptors over time (6 h, 24 h, and 72 h) [126]. Significant changes were observed in all cavitation groups at 6 and 24 h and returned to baseline at 72 h. According to the results, inflammatory responses caused by FUS depend on the cavitation dose of MB, so careful monitoring for MB cavitation will be critically required [126]. It is still unclear how FUS-mediated BBB opening affects the induction of neuroinflammation. Choi et al. recently reported changes in the inflammatory response according to FUS parameters (0.25 MPa and 0.42 MPa) [127]. Although micro-bleeding and tissue damage were observed, the BBB disruption effect was three times higher in the 0.42 MPa-treated group. As a result of transcriptome analysis, the expression level of NF-kB pathway-related genes was regulated in a time-dependent manner only in the 0.42 MPa treatment group. In addition, the induction of neuroinflammation through glial cell activation was confirmed in the 0.42 MPa group, but neuroprotective effects were specified by the expression of A2-type astrocytes. Therefore, the non-excessive 0.25 MPa parameter can control the BBB without a sterile inflammatory response. In addition, when excessive FUS parameters are used, a sterile inflammatory response can be induced through the activation of glial cells, suggesting that A2-type astrocytes affect the homeostasis of the brain microenvironment.

Since immune responses by FUS-mediated BBB opening depend on various factors, including differences in parameters and cavitation dose, inflammatory responses occur at different time points that can be prolonged or quickly vanish. As clinical studies on FUS-mediated BBB opening are actively expanding, we need to better understand varying inflammatory responses affected by FUS.

## 4. Conclusions and Future Directions

This review briefly summarizes how FUS-mediated BBB opening is currently being studied in AD, brain tumors, and PD. Moreover, the neurogenesis or immune response induced when the BBB is opened and the glymphatic system associated with recent clearance were briefly introduced. Although not mentioned in this review, FUS studies are underway in various diseases, such as amyotrophic lateral sclerosis, traumatic brain injury, and stroke. They will be expanded to more diseases in the future.

Currently, FUS is considered an innovative treatment method that effectively treats various neurological disorders that have been challenging to overcome for a long time. The effects and safety of FUS-mediated BBB opening on the brain have already been studied extensively. However, despite many technological advances in FUS over the past 20 years, research on the clinical applications of FUS-mediated BBB opening is only just beginning. Recently, clinical trials of FUS-mediated BBB opening have confirmed its safety in patients with AD [128,129], PD [130,131], and brain tumors [132,133]. However, the exact mechanism of drug delivery by FUS-mediated BBB opening has not yet been elucidated. In addition, there is a need to identify the mechanisms underlying the various biological effects of BBB opening. Furthermore, since various effects of FUS stimulation without MB have been reported, comparative studies of BBB opening with MB are needed. Clinical optimization studies are needed to standardize FUS-mediated BBB opening as a new treatment modality, and preclinical research studies are needed to confirm the clinical effect of this modality.

Among the latest medical technologies being developed for the treatment of many neurological disorders, one of the major directions is a noninvasive or minimally invasive treatment. The main advantage of FUS is that it is a non-invasive technology; therefore, it is relatively safe and can be used repeatedly. FUS may be established as a representative treatment technique for neurological disorders in the near future.

## Figures and Tables

**Table 1:** Recent preclinical studies on focused ultrasound-mediated blood–brain barrier opening in Alzheimer’s disease.

Authors, Year of Publication	Animal Model	FUS Parameters	Target Region	Main Results
Xhima (2020) [25]	TgCRND8 mice	CF:1.68 MHz PRF:1 Hz TD:120 s AP: Maintained after decreasing to 25% based on subharmonic emissions	Basal forebrain	Delivery of D3 (peptidomimetic agonist of TrkA) to the basal forebrain via FUS activated the TrkA-related signaling cascades and increased cholinergic neurotransmission.
Dubey (2020) [28]	TgCRND8 mice	CF:1.68 MHz PRF:1 Hz TD:120 s AP: 0.23 MPa (feedback controller)	Cortex and hippocampus	IVIg-FUS significantly increased neurogenesis. FUS alone and IVIg alone significantly reduced amyloid plaques. IVIg-FUS affects neurogenesis through the downregulation of TNF-α.
Deng (2021) [48]	APP/PS1 transgenic mice	CF:1 MHz PRF:10 Hz TD:60 s AP:0.6 MPa	Posterior 3.5 Lateral 3.5 Ventral 3.5 (mm)	Proved the possibility of extracting exosomes from astrocytes through ultrasonic stimulation. Astrocyte-derived exosome was delivered to the brain after opening the BBB to confirm the amyloid clearance effect.
Feng (2021) [49]	Sprague-Dawley rats Aβ (1–40) injection model	CF:1 MHz TD:60 s AP:0.8 MPa	Hippocampus	As a result of the delivery of MpLXSN-BDNF (modified MB with retrovirus-BDNF) through FUS, cognitive function is improved, and BDNF restores synaptic loss.
Leinenga (2021) [50]	APP23 transgenic mice	CF:1 MHz PRF:10 Hz TD:6 s AP:0.7 MPa	Whole brain	The combined treatment of scanning ultrasound and Aducanumab induced the effect of reducing amyloid plaques in the hippocampus and restored cognitive function.
Poon (2021) [51]	TgCRND8 mice	CF:1 MHz PRF:1 Hz TD:120 s AP:0.28–0.55 MPa	Hippocampi and cortices	FUS-mediated BBB opening treatment three to five times biweekly did not induce neutrophil recruitment or phagocytosis of amyloid plaques.
Sun (2021) [52]	Aged APP/PS1dE9 mice	CF:278 kHz PRF:2 Hz TD:100 s AP:0.33 MPa	Hippocampi	FUS increased the delivery rate of 07/2a mAb (Fc-competent anti-pGlu3 Aβ monoclonal antibody) to the brain by 5.5 times. Co-treatment with FUS and 07/2a mAb induces greater effects on learning and memory recovery and increases synaptic puncta.
Luo (2022) [53]	Kunming mice Aβ1–42 injection model	CF:1 MHz PRF:1 Hz TD:120 s Voltage: 200 mV	Hippocampus	FUS-Gastrodin treatment restored memory and alleviated neuropathology. FUS-Gastrodin reduced Aβ, tau, and P-tau and upregulated BDNF, synaptophysin, and PSD-95 in the hippocampus.
Bathini (2022) [54]	APP/PS1dE9 transgenic mice	CF:278 kHz PRF:2 Hz TD:100 s AP:0.33 MPa	Cortex and hippocampus	07/2a mAb (anti-pyroglutamate-3 Aβ antibody) delivered with FUS resulted in a 5- to 6-fold increase in the brain-to-blood antibody ratio after 4 and 72 h. FUS-07/2a mAb enhanced the immunoreactivity of resident Iba1+ and phagocytic CD68+ microglia.
Bajracharya (2022) [55]	K3 mice (human 1N4R tau)	CF:1 MHz PRF:10 Hz TD:6 s AP:0.5 MPa	Whole brain	Repeated FUS-BBB opening reduces tau inclusions. FUS-BBB opening mediates delivery of RNF5 (tau-specific monoclonal antibody) increase brain uptake and accumulates in unclear cells within the pyramidal layer.
Kong (2022) [42]	5×FAD mice	CF:0.5 MHz PRF:1 Hz TD:120 s AP:0.25 MPa	Hippocampi	Combined therapy of FUS and Aducanumab decreases amyloid deposits, increases neurogenesis, and attenuates cognitive function deficits.

AP, acoustic pressure; BDNF, brain-derived neurotrophic factor; CF, center frequency; FUS, focused ultrasound; MB, microbubble; PRF, pulse repetition frequency; PSD-95, postsynaptic density protein-95; TD, train duration; TH, tyrosine hydroxylase.

**Table 2:** Recent preclinical studies on focused ultrasound-mediated blood–brain barrier opening in Parkinson’s disease.

Authors, Year of Publication	Animal Model	FUS Parameters	Target Region	Main Results
Ji (2019) [72]	C57BL/6 mice MPTP	CF:1.5 MHz PRF:10 Hz TD:60 s AP:0.45 MPa	Striatum and substantia nigra	FUS-Intranasal delivery increased TH immunoreactivity and improved motor control function.
Lin (2020) [73]	Balb/c mice MPTP	CF:1 MHz PRF:10 Hz TD:180 s Voltage:85 V	Substantia nigra	BDNF or GDNF gene delivery through the UTMD system induces a neuroprotective effect. However, combined with the GDNF/BDNF gene delivery it did not produce benefits compared with individually delivering BDNF or GDNF genes.
Yan (2021) [74]	C57BL6 mice MPTP	CF:1 MHz PRF:1 Hz TD:60 s AP:0.24–0.45 MPa	Cortex, striatum, and substantia nigra	Improves therapeutic efficacy by increasing the delivery rate of encapsulated curcumin through FUS.
Yuhong (2022) [75]	C57BL/6J mice MPTP	CF:1 MHz PRF:1 Hz TD:60 s Voltage:100, 150, 200 mV	Striatum	FUS increased the delivery rate of gastrodin, which induces neuroprotective effects, by 1.8-fold. FUS-Gastrodin treatment increased the expression levels of Bcl-2, BDNF, PSD-95, and synaptophysin protein and decreased the levels of caspase-3 in the striatum.
Trinh (2022) [76]	Sprague-Dawley rats	CF:1 MHz PRF:1 Hz TD:120 s AP:0.4 MPa	Striatum and substantia nigra	FUS-induced BBB permeability in the striatum and substantia nigra. SIRT3-myc (viral vector gene therapies for PD) was expressed only in the striatum.

AP, acoustic pressure; Bcl-2, B-cell lymphoma-2; BDNF, brain-derived neurotrophic factor; CF, center frequency; FUS, focused ultrasound; GDNF, glia cell line-derived neurotrophic factor; MPTP, neurotoxin 1-Methyl-4-phenyl-1,2,3,6-tetrahydropyridine; PD, Parkinson’s disease; PRF, pulse repetition frequency; PSD-95, postsynaptic density protein-95; TD, train duration; TH, tyrosine hydroxylase; UTMD, ultrasound-targeted microbubble destruction.

**Table 3:** Recent preclinical studies on focused ultrasound-mediated blood–brain barrier opening in brain tumors.

Authors, Year of Publication	Animal Model	FUS Parameters	Target Region	Main Results
McDannold (2020) [96]	Sprague-Dawley rats F98 glioma	CF:230 kHz PRF:1.1 Hz TD:55 s AP:119–186 kPa	Striatum (Tumor)	It was confirmed that the ExAblate Neuro low-frequency clinical TcMRgFUS system could stably open the BBB in a rat model. Although delivery of irinotecan to the brain was not neurotoxic, it was not effective in prolonging survival or reducing the growth of gliomas.
Curley (2020) [93]	athymic nude mice U87 GBM	CF:1.1 MHz DC:0.5% TD:120 s AP:0.45–0.55 MPa	Striatum (Tumor)	Interstitial fluid transport in brain tumors is increased by FUS. FUS increased the dispersion of directly injected brain-penetrating nanoparticles through tumor tissue by >100%.
Englander (2021) [97]	B6 mice PDGF-B + PTEN−/−p53−/− murine glioma	CF:1.5 MHz PRF:5 Hz TD:120 s AP:0.7 MPa	Pons (Tumor)	FUS increased the delivery rate of etoposide into the tumor site more than five times compared to the control group, but there was no difference in survival rate or inflammation.
Sheybani (2021) [98]	C57BL/6 mice GL261 glioma	CF:1.1 MHz DC:0.5% TD:120 s AP:0.4 MPa	Striatum (Tumor)	[89Zr]-mCD47 (phagocytic immunotherapy) delivery with repeated FUS can significantly constrain tumor outgrowth and extend survival rate.
Ye (2021) [99]	Swiss-Webster mice GL261 glioma	CF:1.5 MHz PRF:5 Hz TD:60 s AP:0.43 MPa	Brain stem (Tumor)	FUS-mediated intranasal delivery increased the delivery rate of anti-PD-L1 antibodies to the brain stem by 4.03-fold.
Chen (2021) [100]	Fisher rats C6 glioma	CF:400 kHz PRF:1 Hz TD:120 s AP:0.81 MPa	Caudate putamen (Tumor)	CD4+ (helper TILs) and CD8+ (cytotoxic TILs) immunogenic responses were significantly increased after 7 days of FUS treatment.
Moon (2022) [101]	BALB/c nude mice U87 GBM	CF:1 MHz PRF:1 Hz TD:60 s AP:1 W/cm^2^	Cerebral hemisphere	Sonosensitive liposome-encapsulating doxorubicin enhances permeability by FUS-mediated BBB opening. The GBM cytotoxicity of IMP301-DC was significantly increased.
Sheybani (2022) [102]	C57BL/6 mice GL261 glioma	CF:1.1 MHz PRF:1 Hz TD:120 s AP:0.4–0.6 MPa	Striatum (Tumor)	FUS-mediated BBB opening in gliomas transiently induces inflammatory effects.

AP, acoustic pressure; BBB, blood–brain barrier; CF, center frequency; DC, duty cycle; FUS, focused ultrasound; GBM, glioblastoma; PRF, pulse repetition frequency; TD, train duration; TILs, tumor-infiltrating lymphocytes.

## Data Availability

All data listed in this review article are publicly accessible on PubMed.
